# Findings from transcriptomics and immunohistochemistry indicate an autoimmune disease targeting brainstem inhibitory interneurons in bovine spastic paresis

**DOI:** 10.1371/journal.pone.0324633

**Published:** 2025-05-29

**Authors:** Frederik Krull, Shahrbanou Hosseini, Martina Bleyer, Bertram Brenig

**Affiliations:** 1 Div. of Molecular Biology of Livestock and Molecular Diagnostics, Georg-August University Göttingen, Institute of Veterinary Medicine, Göttingen, Germany; 2 German Primate Center, Pathology Unit, Leibniz Institute for Primate Research, Göttingen, Germany; National Center for Toxicological Research, UNITED STATES OF AMERICA

## Abstract

Bovine spastic paresis (BSP) is a progressive neuromuscular disease of unknown origin that causes persistent stiffness of the hind limbs. The symptoms are similar to those of human motor neuron diseases such as primary (PLS) or amyotrophic lateral sclerosis (ALS). BSP occurs worldwide in cattle production with an estimated prevalence of <1%. For Germany, this means that around 20,000 Holstein cattle are affected. BSP is generally considered a hereditary disease, but there is no prevention through breeding programs. As a result, BSP not only affects animal welfare but also leads to economic losses in milk and beef production. Here, we used transcriptomics to analyse the brainstem, spinal cord and affected gastrocnemius muscle tissue of eight animals affected by BSP and eight control animals from slaughterhouses to gain new insights into the molecular mechanisms underlying BSP. We found that the expression of several genes was significantly different in animals affected by BSP compared to control animals. Specific genes for inhibitory neurons were downregulated in the brainstems of the affected animals, namely *CCK* (cholecystokinin), *NPY* (neuropeptide Y), and *SST* (somatostatin). These inhibitory neurotransmitters influence cerebral movement control, among other processes. Furthermore, *OOSP2* (oocyte secreted protein 2) was found to be significantly upregulated in the affected animals in all tissues. This expression could best be explained by the presence of T-follicular-helper cells which, through interleukin 21, can trigger a TH-2-dominated immune response and lead to autoimmune encephalitis. Further cases were sampled for confirmation and we detected cell infiltrates of activated microglia and T-cells in the brainstem using immunohistochemistry. Microglial foci were significantly more abundant in animals affected by BSP than control animals. We conclude that BSP is caused by an autoimmune reaction directed against inhibitory interneurons in the brainstem and is due to a combination of genetics and environmental influences. This may result in lost controlling influence on the upper motor neurons via extrapyramidal pathways and therefore triggers the specific symptoms of motor neuron disease.

## Introduction

Bovine spastic paresis (BSP) was first described over a century ago, but its causes are not yet fully understood. BSP is thought to be a hereditary and degenerative disease of the nervous system, but there is a lack of experimental evidence to support this [1,2]. BSP occurs as an early onset form in calves at the age of a few weeks or as a late onset form in adults at the age of about two to seven years [3]. Spasms worsen over time and with progression, both limbs may be affected [2]. Affected animals show isolated, protracted spasms of the gastrocnemius muscle (BSP-G) or the quadriceps muscle (BSP-Q), or a combination of both with involvement of other hind limb muscles (BSP-M). The only cure for BSP-G is symptomatic and involves the neurectomy of specific branches of the tibial nerve responsible for innervating the gastrocnemius muscle. There are currently no causal therapies available, as target genes have not yet been identified [3,4]. The prevalence of BSP in German Holstein cattle has been estimated at 0.54%, but the disease has been described worldwide and in almost all cattle breeds [5,6]. There is no information in the literature about the prevalence of BSP in other regions of the world.

The primary differential diagnosis for BSP is bovine spastic syndrome (BSS), but in BSS tremor is the main symptom rather than spasm, and no early onset of the disease is described [7]. BSP-like symptoms have also been described in goats and pigs, but no corresponding genes have been detected in these species [8,9]. BSP-like encephalitis has been reported in several mammalian species, including donkeys, cats, bats, kangaroos, and capybaras, due to Rustrela virus infections [10,11].

Knowledge of the mechanisms of spastic diseases in cattle is limited, but the main symptoms of BSP, i.e., isolated rigidity without a tremor, occur similarly in several human motor neuron diseases. Consequently, BSP can be used as a disease model for primary lateral sclerosis (PLS), amyotrophic lateral sclerosis (ALS), and hereditary spastic paraplegia (HSP) [12–14]. These progressive, neurodegenerative diseases are the subject of intensive research and are studied in different animal models, including mice, non-human primates and zebrafish models [15,16]. PLS is symptomatically most similar to BSP and is a degenerative disease of the upper motor neurons for which causative genes have not been identified. It manifests as unilateral spasms of the hind limbs and tongue and occurs in a late- and early-onset form (juvenile primary lateral sclerosis) [17,18]. ALS is caused by a combined degeneration of both motor neurons and leads to a dramatic symptom complex with weakness and severe muscle wasting [19]. Like BSP, ALS and various forms of hereditary spastic paraplegia occur in either young or elderly patients and cause similar symptoms, with over 100 target genes described [20,21].

The activity of upper motor neurons is decisively influenced by networks of excitatory and inhibitory neurons in the brainstem [22]. Degeneration of the upper motor neuron or corticospinal tract leads to a lack of inhibitory control of movement by the brain, resulting in an uncontrolled release of acetylcholine and permanent contraction of the innervated muscles [17]. Degeneration of the lower motor neuron leads to flaccid paralysis with severe muscle wasting [19].

The following four molecular pathological mechanisms have been identified as the primary causative factors of neurodegeneration in the aforementioned diseases: I) The transport of neurotransmitters in vesicles along the axons to the synapses or the packaging of the neurotransmitters in vesicles at the endoplasmic reticulum may be impaired [21]. II) Oxygen radicals can be released from the mitochondria or the formation of adenosine triphosphate (ATP) can be impaired [21]. III) The synthesis of neurotransmitters is impaired [21]. IV) Immune processes can destroy neurons or leukodystrophy can destroy the myelin sheaths of the axons, leading to neurodegeneration [23].

In this study, we used mRNA shotgun sequencing and immunohistochemistry to search for the affected metabolic pathways and identify possible pathological changes responsible for BSP in Holstein cattle.

## Materials and methods

### Ethics

Tissue samples were collected at German abattoirs. The collection of samples was approved by the Lower Saxony State Office for Consumer Protection and Food Safety (33.19-42502-05-17A196) according to §8a Abs. 1 Nr. 2 of the German Animal Protection Law.

### Sample collection

In the federal state of Lower Saxony (Germany), a Holstein breeding farm was identified in which about 10% of the offspring showed BSP symptoms at the age of 12 months. The animals were photographed and filmed to document the signs. The animals were also clinically examined to ensure that they were suffering from BSP before samples were taken. Tissue samples of the gastrocnemius muscle, the spinal cord (lumbar region L1-L4), and the brainstem (medulla oblongata, pons, mesencephalon) were taken with a sharp spoon at the slaughterhouse from a total of eight animals suffering from BSP and eight control animals. Tissue samples were collected at two abattoirs, with two months between visits. In each experimental group (case/control), two animals were male and six animals were female. At the time of slaughter, all animals were between 12 and 20 months old. Tissue samples from all individuals (8 cases, 8 controls) were preserved in RNAprotect Tissue Reagent (Cat# 76104, Qiagen, Hilden, Germany) immediately after dissection and stored at −80°C for RNA-Seq analysis.

**Fig 1:**
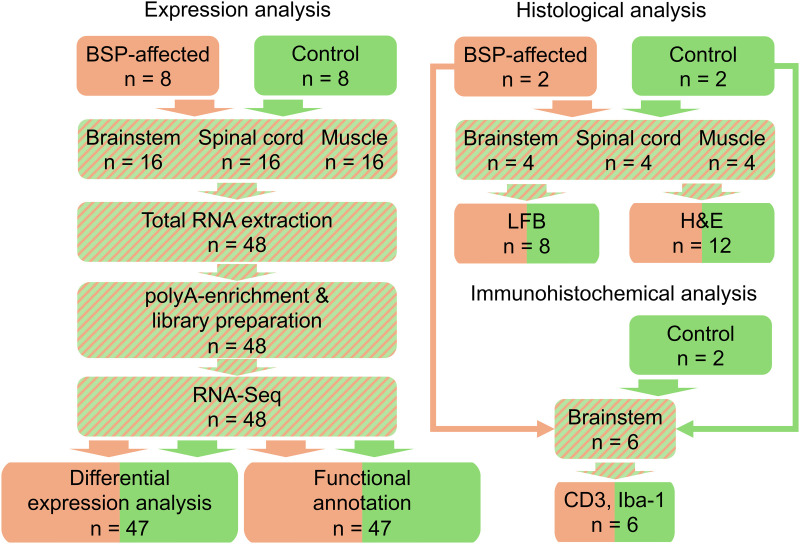
General overview of the experimental design. RNA-Seq analysis of tissue from the brainstem, spinal cord, and gastrocnemius muscle in a case/control design. The cohorts consisted of eight BSP-affected cases and eight controls. Subsequent histological examinations on additional samples for phenotypic validation of the RNA-Seq results showed mild pathological changes in the brainstem in both cohorts. For quantification of inflammatory cells, immunohistochemistry (CD 3 and Iba-1) was performed on four brain stem sections of each individual.

Further tissue samples from two cases and four controls from two other abattoirs were taken for histological analysis. The dissected samples were fixed in 10% formalin (Avantor, Leuven, Belgium) and stored at 4°C for histological analysis. An overview of the experimental setup, sampling, and laboratory analyses of this study is shown in Fig 1.

### Immunohistochemical analysis

The formalin-fixed tissue samples were manually cut into smaller pieces, embedded in paraffin, and cut with a microtome to obtain 5 µm tissue sections, which were automatically stained with haematoxylin and eosin (H&E). Sections of the brainstem and spinal cord were additionally stained with luxol-fast-blue (LFB) [24]. Immunohistochemistry (IHC) was performed on paraffin-embedded sections of the brainstem using an anti-CD3 antibody labeling T-cells (Cat# A0452, Dako Deutschland GmbH, Hamburg, Germany, polyclonal rabbit anti-human CD3, 1:50) and an anti-Iba-1 antibody to visualize the macrophage/microglia-specific calcium-binding protein Iba-1 (Cat# 100042, GeneTex, Biozol Diagnostica Vertrieb GmbH, Eching, Germany, rabbit polyclonal, 1:100). For each animal, 4 sections of brainstem with intervals of 250 µm between sections were stained with both antibodies. IHC was performed with an automated immunostaining system (Discovery XT, Roche Diagnostics GmbH, Mannheim, Germany) using the SABC (Streptavidin-Biotin-Complex) method, DAB (diaminobenzidine tetrahydrochloride) for signal detection (DAB Map Kit, Cat# 760−124, Roche Diagnostics GmbH, Mannheim, Germany) and hematoxylin for counterstaining. Rhesus macaque lymph node and brain tissue previously confirmed to show cross-reaction with both antibodies (according to in-house validation) served as positive controls. Pure antibody diluent was applied to the sections of the negative control. The sections were examined microscopically for pathological changes. If present, findings were documented descriptively. In addition to the qualitative evaluation of the sections, a quantitative analysis of CD3-positive cells and microglial activation was performed to assess possible differences in the distribution of Iba-1-expression and in the number of CD3-positive cells between the affected animals and the controls. Therefore, IHC slides were scanned with an Aperio CS2 slide scanner at a maximum magnification of 20 x (Leica Biosystems GmbH, Nussloch, Germany). Digital images (.svs files) were uploaded into a QuPath project (version 0.4.3). After the image type setting (Brightfield_H_DAB), color deconvolution for stain 1 (hematoxylin) and 2 (DAB) was manually performed on each image using a small rectangle region of interest (ROI) in the image followed by the auto-detection of stain vectors (set by estimate stain vectors) [25]. For quantification of CD3 positive cells, ten rectangles with an area of 2250000 µm^2^ (1500 x 1500 µm) were randomly applied to each section (4 sections of brainstem per animal) using the command classify > training images > create region annotations and selecting “random”. As squares were placed anywhere in the image including the background area, the command was repeated until 10 squares were created that were all located within the tissue area without overlap and leaving out areas with extensive artifacts (e.g., folds). CD3-positive cells were then counted manually in the 10 rectangles per section. For quantification of microglial activation in the brainstem, an annotation (region of interest = ROI) was set for each section using the classify > pixel classification > create thresholder command as a first step. Thresholds were individually set for each section to create an annotation (ROI in µm^2^) that fully encircled the brainstem tissue. In a second step, foci with increased and condensed Iba-1-expression with a minimal diameter of 100 µm were identified and their total number was counted manually within the respective ROI [25]. Manual counting results were statistically analysed with Students-T-Test and considered unevenly sized, unpaired, and independent cohorts.

### RNA-Seq and genome alignments

Tissue samples were thawed and approximately 100 mg of each brainstem and spinal cord sample and 50 mg of each muscle sample was dissolved in QIAzol Lysis Reagent (Cat# 79306, Qiagen, Hilden, Germany) in a FastPrep FP120 Homogeniser (Thermo Fisher, Waltham, MA, USA) and total RNA was extracted using the RNeasy Plus Universal Mini Kit (Cat# 74136, Qiagen, Hilden, Germany). The concentration and integrity of the extracted RNA (RIN) was measured on an Agilent RNA 6000 Nano Chip using an Agilent 2100 Bioanalyzer (Cat# 5067−1511, Agilent Technologies, Santa Clara, CA, USA). RNA samples were poly(A) mRNA enriched using the NEBNext Poly (A) mRNA Magnetic Isolation Module (Cat# E7490, NEB, Frankfurt, Germany). Enriched poly(A) mRNA was transcribed into cDNA using the NEBNext Ultra II RNA Library Prep Kit for Illumina. Adaptor ligation and indexing was done with NEB Multiplex Oligo Kit for Illumina (Cat# E7335, NEB, Frankfurt, Germany). Each reaction step was conducted on a Biometra T3000 thermocycler (Biometra, Göttingen, Germany).

Library amplification was done in a Roche 480 Lightcycler (Roche Diagnostics GmbH, Mannheim, Germany) with EvaGreen (Cat# 379, Jena Bioscience GmbH, Jena, Germany) as a dye. The quality of the libraries was assessed using DNA 7500 Chips on an Agilent 2100 Bioanalyzer (Cat# 5067–1506, Agilent Technologies, Santa Clara, CA, USA). The libraries had an average concentration of 491 ng/μl with RINs of 3.7–7.8. Four nM of each library were pooled and applied to a flow cell for PE75 sequencing on a NextSeq 500 System (Cat# 20024906, Illumina, San Diego, CA, USA). An average number of 17.5x10^6^ reads per sample was obtained.

The quality assessment of raw sequencing data (fastq files) was performed using FastQC version 0.11.4 [26] and MultiQC version 1.13 [27]. The fastq files were trimmed using Trimmomatic version 0.36 [28] to remove adapters, low quality bases, and low-quality reads. One BSP brainstem sample was removed from further analysis due to low quality. The cleaned sequences were then mapped to the *Bos taurus* reference genome (ARS-UCD1.2, GCA_002263795.2) downloaded from the Ensembl database [29] using the program STAR 2-pass mapping version 2.7.3a [30]. Finally, FeatureCounts version 2.0.0 [31] was used to count the number of reads mapped for each gene across all samples. All software packages for bioinformatics analysis were run using the standard settings.

### Differential gene expression analysis

**Fig 2:**
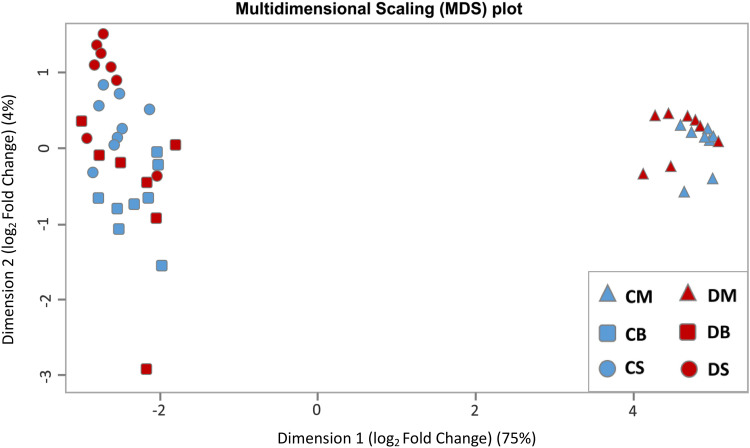
Multidimensional scaling plot of the RNA-Seq data. After principal component analysis of the RNA-Seq data, the x-axis represents the first dimension, explaining 75% of the overall variance in the dataset. The y-axis represents the second dimension, explaining 4% of the overall variance. Experimental groups are encoded as follows: blue triangle = control muscle, red triangle = disease muscle, blue square = control brainstem, red square = disease brainstem, blue circle = control spinal cord, red circle = disease spinal cord. Mind the appropriate admixture in cases and controls within tissues and larger differences within investigated tissues.

Differentially gene expression analysis in this study was performed with the program R-Statistics using the “edgeR” package [32,33]. For this analysis, only genes with more than 1 CPM (counts per million reads) across all samples of at least one condition (control, disease) were considered to avoid unreliable results. This resulted in a matrix of 16,088 genes for 47 samples with 8 biological replicates per experimental group: control brainstem, disease brainstem, control muscle, disease muscle, control spinal cord, and disease spinal cord, except for disease brainstem with 7 biological replicates, as explained above. For each tissue (brainstem, muscle, and spinal cord), disease groups (BSP-affected groups) were compared with control groups to identify genes that are differentially expressed between two different conditions. In general, the analysis of differential gene expression was performed in three main steps: (1) normalising gene counts using sequence depth for sample-specific effects; (2) applying a generalized linear model (GLM) which underlies a negative binomial distribution; (3) performing the likelihood ratio test to compare whether gene expressions differed significantly between two conditions [34]. Fig 2 shows the multidimensional scaling plot to help determine the overall differences between cases and controls and variability within cases and controls.

To account for multiple testing correction, the standard Benjamini-Hochberg false discovery rate (FDR) approach was used [35], in which the expression differences between the different experimental groups were considered to be statistically significant at an FDR of < 0.05. To further investigate the significantly differentially expressed genes detected in this study, a list of candidate genes associated with human HSP was compiled. The list was collected from a review paper on human HSP and a gene set from a previous microarray-based expression study in spinal cord tissue from BSP-affected Romagnola cattle [6,21]. Due to the low quality of genetic associations in the literature for cattle, we additionally used human HSP as a model disease. A total of 248 genes were selected as candidates (S1 Table).

### Validation of expression differences with digital droplet PCR

To confirm the significant differences in gene expression for the four most interesting genes, i.e., *CCK*, *NPY*, *SST*, and *OOSP2*, TaqMan-based digital droplet PCR (ddPCR) assays were designed [36]. The sequences of the primers and probes used as well as the PCR protocols can be found in the Supplementary Data (S2 Table). Therefore, additional RNA extracts were isolated from all tissues and each sample as described above. These extracts were converted to cDNA using the Maxima H Minus First Strand cDNA Synthesis Kit (Cat# K1681, ThermoFisher Scientific, Dreieich, Germany). Individual ddPCRs were performed for each cohort and tissue using pooled cDNA extracts. Droplets were generated in a QX200 droplet generator and analyzed after PCR using the QX200 droplet reader and QuantaSoft software (Bio-Rad Laboratories GmbH, Feldkirchen, Germany).

### Functional annotation analysis

The significantly differentially expressed genes (FDR < 0.05) were used for gene set enrichment analysis to gain insight into their biological interpretations. All functional enrichment analyses were performed using the R-Statistics program. For gene ontology (GO) analysis using the GO database [37], the topGO package version 2.50.0 [38] was used together with the bovine genome annotation “*org.Bt.e.g.,db*” version 3.8.2 [39] to test the enrichment of GO terms describing biological processes, cellular components, and molecular functions by applying Fisher’s Exact Test. The GOs with *p*-values < 0.05 were considered enriched in the gene set enrichment analysis (S1 Fig). For pathway analysis, the clusterProfiler package version 4.6.0 [40] was applied together with the bovine genome annotation “*org.Bt.e.g.,db*” version 3.8.2 [39] using the default setting in terms of gene set size and *p*-value to test the enrichment of the activated or suppressed pathways for each gene set in the Kyoto Encyclopaedia of Genes and Genomes (KEGG) database [41]. Due to the limited number of animals and significantly differentially expressed genes, we are aware that our test might be underpowered.

### Sequence data accession numbers

RNA-seq data from this study have been submitted to NCBI under accession number PRJNA1151228.

## Results

### Clinical signs of BSP-affected animals

**Fig 3:**
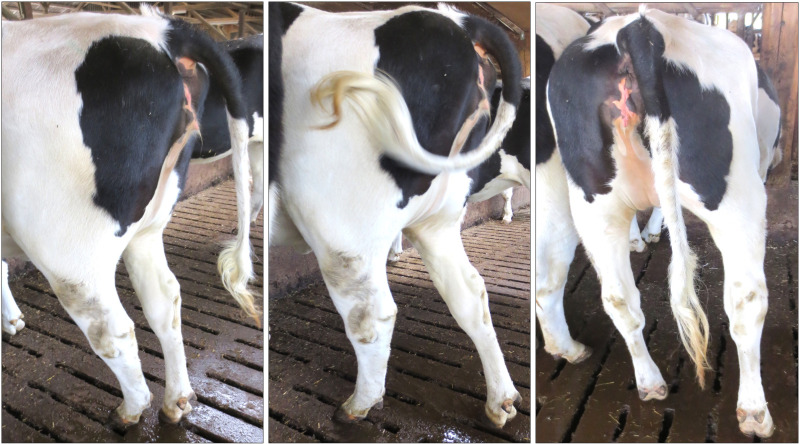
Clinical signs of bovine spastic paresis. Images of a 12-month-old calf affected by BSP, with one leg persistently hyperextended at the ankle joint. Note the straight hocks, the slightly atrophied musculature of the limb, and the lateral deflection of the tail.

Clinical examination of six approximately one-year-old animals revealed caudal flexion of one leg due to a spasm that lasted longer than one minute. This led to hyperextension of the tarsal joint. The tail root was lifted and deflected towards the affected leg. The animals showed no growth retardation. Fig 3 shows photographs of the clinical signs of an affected calf. The movement of the leg was restricted with evidence of rotation. The examined animals showed typical signs of early-onset BSP. Older animals in the herd were also affected. A video of two dairy cows is provided in S2 Video. Babinski reflexes according to the methods of human medicine could not be induced [42].

### Histological and immunohistochemical analyses

**Fig 4:**
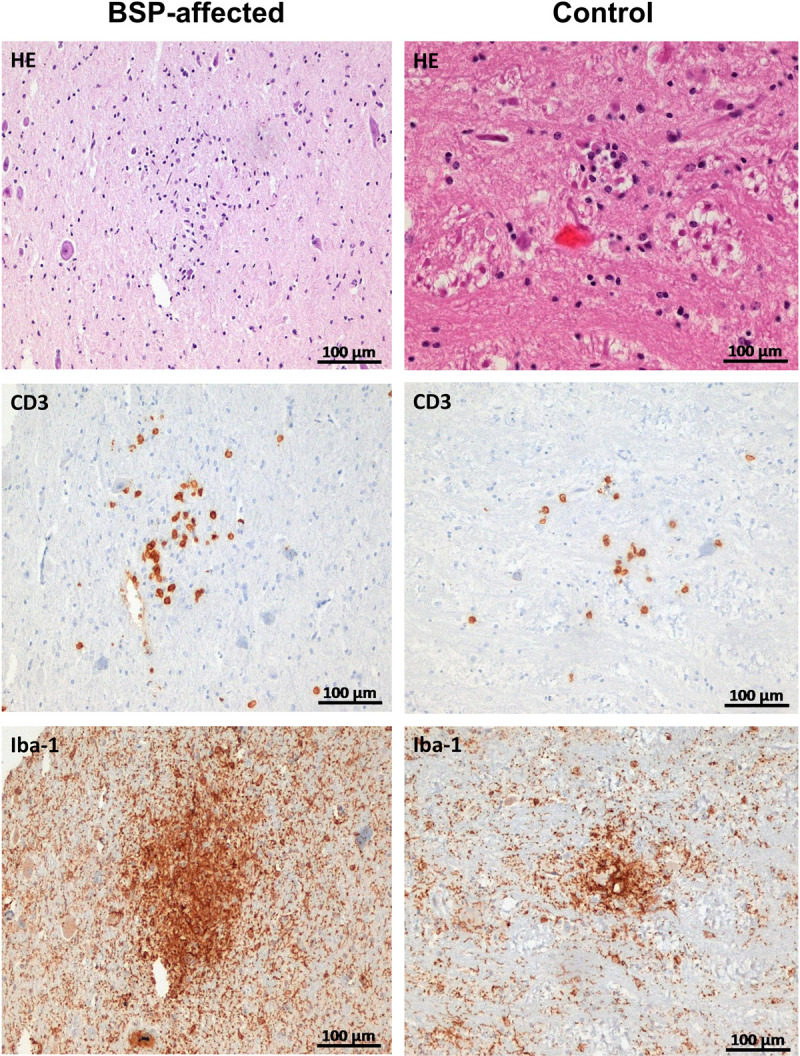
Representative images of the histology of the brainstem. H&E staining shows cellular infiltration in the neuronal tissue. CD3 stained sections show infiltrates of T-cells detectable in cases and controls. Tissue sections stained with Iba-1 show foci of activated microglial cells, in both cohorts. Infiltrates are more pronounced in the affected animals than in the controls.

Histological evaluation of H&E-stained tissue sections of the brainstem showed multiple decent foci of increased cellularity in an animal affected by BSP. In this animal, immunohistochemistry on 4 brainstem sections revealed multifocal clusters of CD3-positive T lymphocytes consisting of at least 20 T-cells, as well as multiple foci of activated microglia visualised by condensed Iba-1 expression of varying size but with a minimum diameter of 100 µm. The distribution of CD3-positive lymphocytic infiltrates and activated microglia appeared randomly distributed and partially angiocentric, affecting both areas with fiber tracts and regions with nuclei. In the second BSP case, there were only a few comparable foci of T-cell accumulation or microglial activation in the 4 immunohistochemically stained brainstem tissue sections. These were rather discrete and hardly visible in the corresponding H&E stained sections. In the brainstem sections of the control animals, however, there was also occasional evidence of lymphocytic infiltration and microglial activation. Fig 4 shows representative histological images of brainstem tissues.

To obtain an indication of whether the described brainstem lesions were coincidental or disease-related, quantification of T-cells was performed, resulting in a total number of 675 T-cells in 80 rectangles in the 2 BSP-positive animals and 1104 T-cells in 160 rectangles in the 4 control animals. Quantification of activated microglia revealed 55 foci in a total of 8 sections of the brainstem in the BSP-affected animals and 24 foci in a total of 16 sections in the control animals. Using Student’s t-test, there was a significant difference in microglial activation between BSP-affected and control animals (p = 0.002157, t = 3.18). A statistically significant difference was not detected in CD3-positive T-cells between the cohorts. However, there was a trend towards a higher number of T-cells in BSP-affected animals (p = 0.09103, t = 1.34). Table 1 shows the counting results of the IHC sections. Other histological findings including multifocal perivascular hemorrhages in the brainstem in BSP affected and control animals were interpreted as artefacts caused by the slaughter process. Demyelination was not detected by LFB staining in any BSP case or control animal. Microscopic examination of H&E-stained sections of skeletal muscle and spinal cord did not reveal specific pathological findings.

**Table 1:** Counting results of quantitative immunohistochemistry for foci of activated microglia in brainstem samples according to Iba-1 staining and foci of T-cell infiltrates according to CD3 staining.

Cohort	Slide name	No. of foci with T-cell infiltrates (manually encircled + counted)	No. of foci with microglia activation (manually counted in random squares)
Case	K 2920-1A	7	8
Case	K 2920-1B	10	21
Case	K 2920-1C	8	9
Case	K 2920-1D	4	7
Case	K 2921-1A	3	4
Case	K 2921-1B	1	4
Case	K 2921-1C	0	0
Case	K 2921-1D	2	2
Control	K 2922-1A	0	1
Control	K 2922-1B	0	1
Control	K 2922-1C	0	1
Control	K 2922-1D	0	1
Control	K 2923-1A	0	0
Control	K 2923-1B	0	2
Control	K 2923-1C	0	3
Control	K 2923-1D	0	2
Control	K 2937-A	1	0
Control	K 2937-B	2	1
Control	K 2937-C	0	1
Control	K 2937-D	1	0
Control	K 2938-A	4	7
Control	K 2938-B	3	2
Control	K 2938-C	1	1
Control	K 2938-D	2	1

### Differentially expressed genes in tissues of the neuromuscular axis

**Fig 5:**
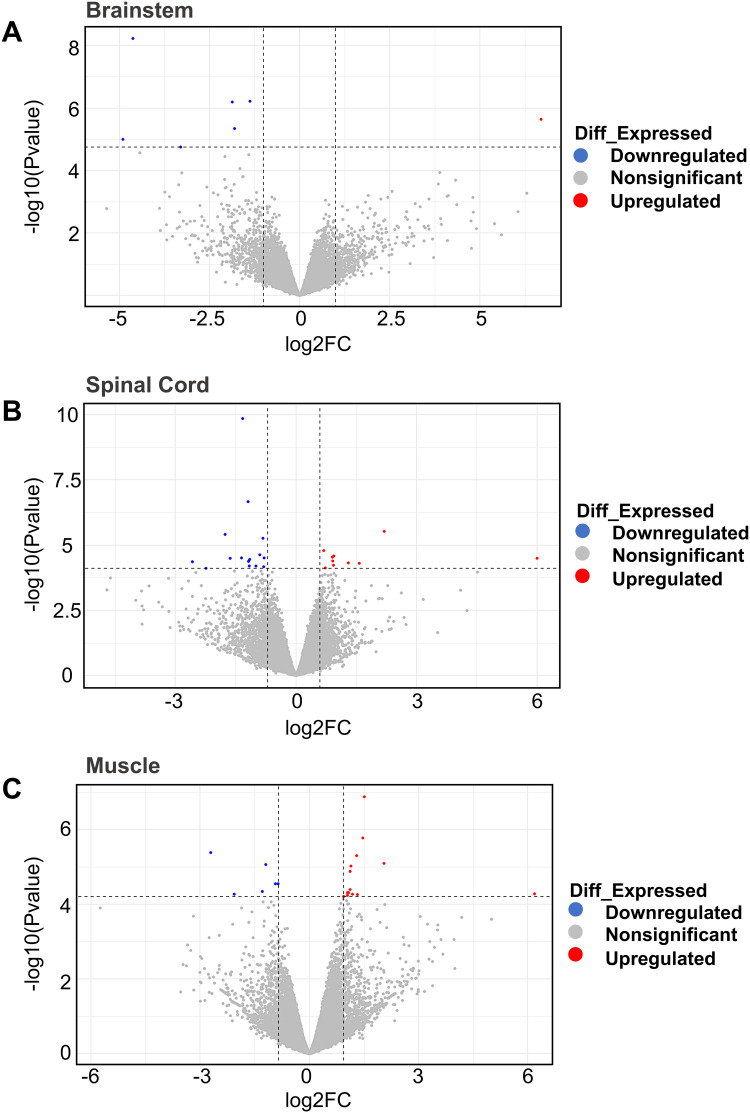
Overall distribution of differentially expressed genes. The volcano plots illustrate differentially expressed genes in BSP-affected cases versus control animals in the respective tissues, brainstem (A), spinal cord (B), and muscle (C). Each dot in the plots represents a gene with its corresponding log_2_ fold change (FC) on the x-axis and −log_10_ (*p*-value) on the y-axis. Red color dots show significantly upregulated genes in the BSP-affected and blue color dots represent significantly downregulated genes. The significant expression differences are shown at the significant threshold (FDR < 0.05). The top 5 FDR-significant DEGs in each tissue are labeled in ascending order.

Samples from the brainstem, spinal cord, and gastrocnemius muscles were analysed for differential expression compared to a control cohort to quantify genetic transcription in tissues of the neuromuscular axis in BSP-affected cattle. The results showed a low number of significant differentially expressed genes (DEG) in each tissue and are shown in Fig 5.

**Fig 6:**
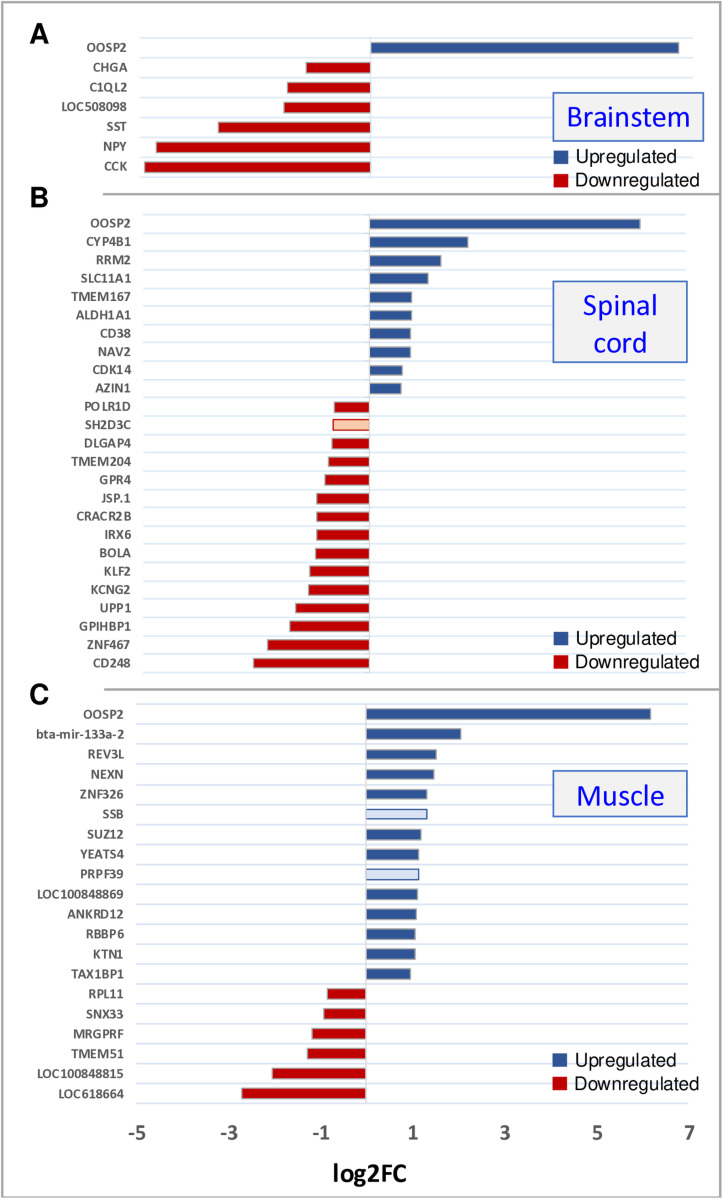
Level of significantly differentially expressed genes (DEGs). The bar charts illustrate the up- and downregulation of significant DEGs in BSP-affected versus control animals at the significant threshold (FDR < 0.05) in the brainstem (A), spinal cord (B), and muscle (C) tissues. The identified significantly differentially expressed candidate genes are highlighted. The x-axis represents the differences in mean fold change (log2) per gene and the y-axis represents the names of significant DEGs.

In the brainstem, only one gene, *OOSP2* (oocyte-secreted protein 2), was significantly upregulated in cases compared to controls. The genes for cholecystokinin (*CCK*), neuropeptide Y (*NPY*), and somatostatin (*SST*) were significantly down-regulated, along with three other genes. In spinal cord tissue, ten genes were upregulated and fifteen genes were downregulated. In muscle tissue, fourteen genes were up- and six genes downregulated. Fig 6 shows all significant expression differences between cases and controls. Significant expression differences in isoforms of genes between cases and controls were not detected in any of the analysed tissues.

**Fig 7:**
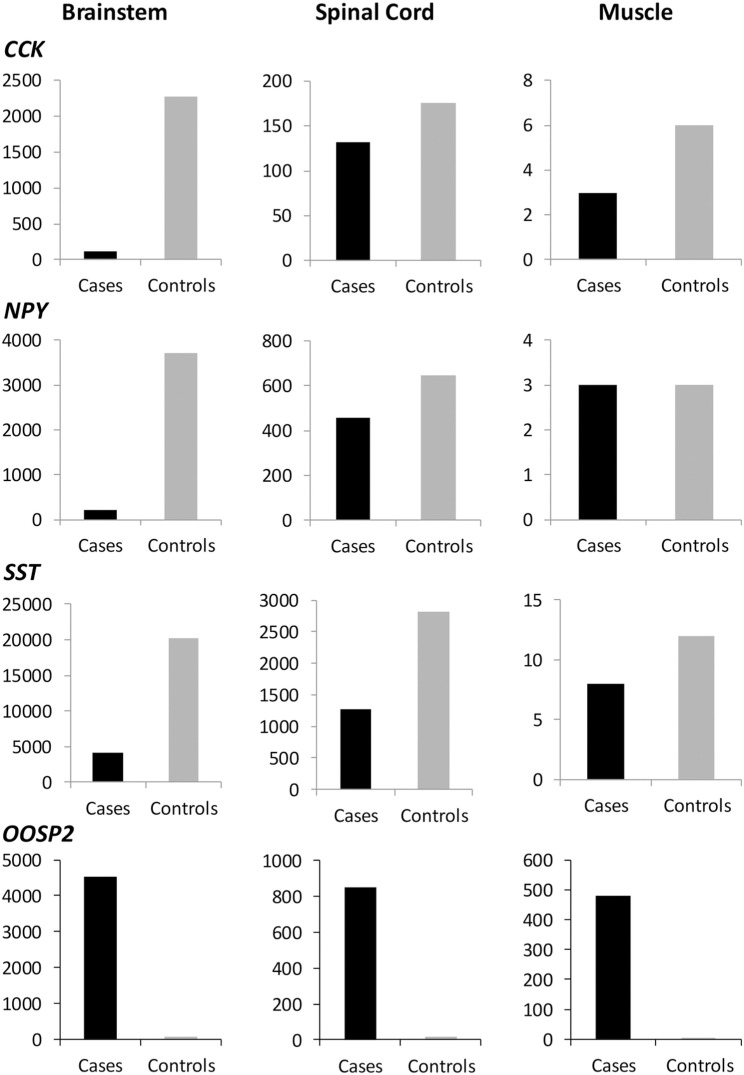
DdPCR results after Poisson correction. Histograms of transcriptional differences in cases and controls in ddPCR for all three tissues analysed for four candidate genes. The histograms on the left show expression differences in the brain stem, in the center in the spinal cord, and on the right in the muscle tissue. The histograms at the top show the copy numbers for the *CCK* gene, below *NPY*, below *SST*, and below *OOSP2*. The Y-axes show the absolute copy numbers per 20µl after Poisson correction. The X-axes distinguish between case and control cohorts. Note that all relevant neurotransmitters are significantly reduced in the brainstem of the cases, while *OOSP2* is upregulated in all tissues.

To validate expression differences, we used ddPCR and applied Poisson-corrected counts for Pearson’s chi-squared tests. Significant expression differences between the BSP cohort and controls were found for all four genes analysed in the brainstem and spinal cord tissue (p < 0.001), this difference is less clear for *CCK* in the spinal cord, but still significant (p < 0.05). For the three neuronal transmitters (*CCK, NPY*, and *SST*), the differences in muscle tissue were not significant. For *OOSP2*, however, the differences in expression were significant in all three tissues. Fig 7 shows the histograms of the results from the ddPCR experiment.

### Gene candidates associated with bovine spastic paresis

The candidate gene list contains a collection of genes that were found to have significantly different expression levels in a study of spinal cord tissue from BSP-affected Romagnola cattle [6]. Three genes from this list also showed differential expression in the study conducted here. One of them, *SH2D3C* (SH2 domain-containing protein 3C), was significantly downregulated in the spinal cord tissue both here and in the above-mentioned study. The other two genes, *SSB* (Lupus La protein homolog) and *PRPF39* (pre-mRNA processing factor 39), were significantly upregulated in the muscle tissue here but downregulated in the spinal cord tissue of the “Romagnola” study.

**Fig 8:**
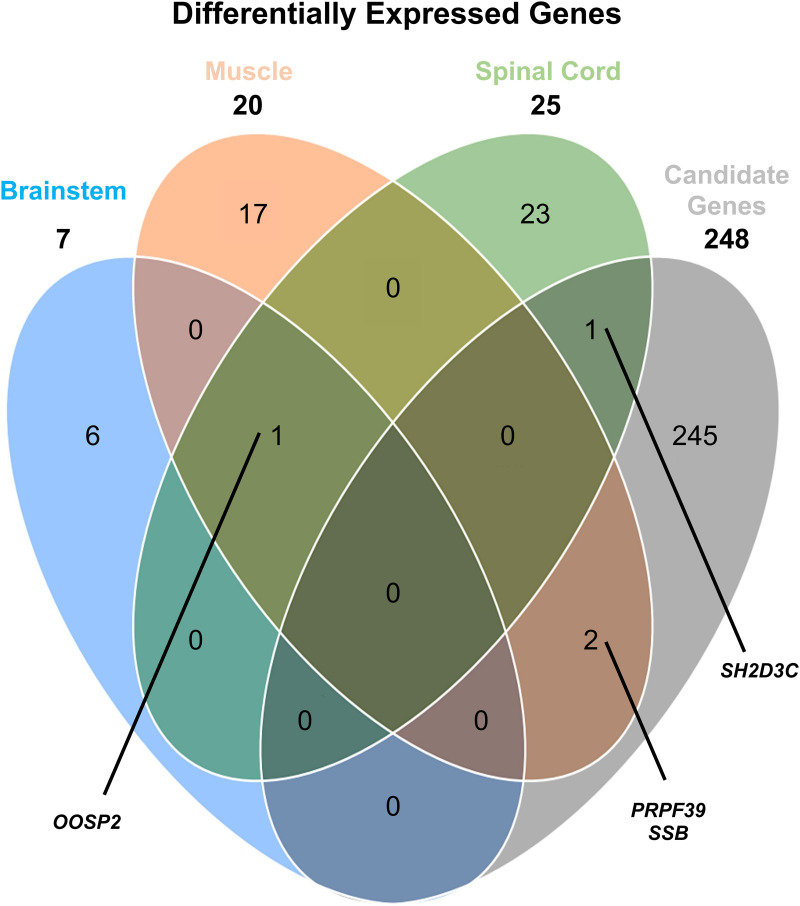
Venn diagram of significantly differentially expressed genes (DEGs). The Venn diagram represents the number of uniquely expressed genes in BSP-affected versus control animals, with the overlapping regions illustrating the number of genes co-expressed in the three different tissues analysed and the candidate genes used in this study. Co-expressed genes are labeled by gene symbols.

No differential expression in the spinal cord tissue was identified here. Although *OOSP2* is not included in the list of candidate genes, it was significantly overexpressed in all tissues here. It was the only gene with significant expression differences in multiple tissues and provided the clearest signal in each tissue. Fig 8 shows common DEGs in the three tissues analysed and representatives from the list of candidate genes.

### Functional annotation of differentially expressed genes

The significant DEGS of all three tissues were subjected to a functional enrichment analysis and categorised into Gene Ontology (GO) categories. In this way, a total of 21 significant (p < 0.05) GO terms were identified which were related to biological processes, four to cellular components, and three to molecular functions in the brainstem. Analysis of functional enrichment in the spinal cord revealed 15 significant GO terms related to biological processes, three related to cellular processes, and three related to biological functions. In muscle tissue, we identified a total of nine significant GO terms related to biological processes, one related to cellular components and six related to molecular functions. The complete lists of enriched GOs can be found in S3 Table. In all tissues analysed, the GOs for cellular components represented membranous and vesicular structures. In brain tissue, seven GOs for biological processes represented terms related to the immune system. GOs for molecular functions related to receptor binding and activation were represented in all three tissues. Pathway analysis revealed enrichment of one pathway in the brainstem (neuroactive ligand-receptor interaction), by *CCK* and *NPY*, which were downregulated in the cases. There were no pathways enriched by the DEGs in the spinal cord and muscle tissue.

## Discussion

Due to the increasing number of cases of bovine spastic paresis (BSP) worldwide, there is an urgent need to clarify the cause of this disease and thereby possibly curb its further spread through breeding. BSP is a widespread neuromuscular disease. Although it results in relatively clear clinical signs of persistent stiffness of the hind limbs, the exact mechanism that triggers this process remains unclear. Several genomic association studies have been performed with conflicting results, so the exact mode of inheritance or penetrance of the genetic variants is still unknown [4,43]. A causative gene for BSP has not yet been identified and potentially associated genes described so far do not explain all aspects of BSP [4]. However, based on the studies to date, a purely structural cause for BSP seems rather unlikely. Initial expression analyses of spinal cord samples revealed 268 DEGs [6]. The authors concluded that BSP is probably caused by impaired glycinergic synaptic transmission and alterations in calcium signaling proteins. To further elucidate the pathogenesis of BSP in this study, a transcriptome analysis was performed on tissue samples from the brain stem, spinal cord, and muscles of BSP-affected and healthy control cattle. The selection of these three tissues deliberately included all components involved in the pathogenesis, i.e., nuclear and axonal compartments of the upper and lower motoneurons as well as the neuromuscular junction and effector muscle tissue including all synaptic connections. However, it should be noted that both the choice of methods and the collection of sample material at the abattoir only capture the current temporal and spatial state of the tissue. However, batch effects do not appear to have had a significant impact due to the results from principal component analysis and the limited number of DEGs ultimately identified. The RNA-Seq data did not reveal distinct mRNA isoforms or sequence variants in coding regions associated with BSP which is in line with previous studies.

However, differences in expression were detected in all three tissues as shown in Fig 6. Of particular interest were five downregulated transcripts in the brainstem, which could be functionally linked to the development of BSP, i.e., *CHGA, C1QL2, SST, NPY*, and *CCK. CHGA* is a glycoprotein on secretory granules of most (neuro)endocrine cells and neurons. It regulates the storage and secretion of hormones and neuropeptides [44]. *C1QL2* is mainly expressed in the red nucleus of the brainstem (RN) and is involved in limb control. A deficiency of the encoded protein adiponectin I specifically impairs fine motor coordination [45]. Somatostatin (*SST*), neuropeptide Y (*NPY*), and cholecystokinin (*CCK*) are neuropeptides that influence cerebral motion control, whereby SST has inhibitory effects on motor functions in the brainstem by modulating dopaminergic and GABAergic systems [46]. *NPY* has inhibitory and neuroprotective effects that suppress inflammation in the central nervous tissue. The involvement of this gene in motor neuron diseases has been repeatedly demonstrated in previous studies [22,47,48]. *CCK* is a pivotal molecule in the entire mammalian brain that influences motoric functions by modulating dopaminergic, GABAergic, and serotoninergic activity [49]. *SST* and *NPY* are exclusively detected in inhibitory neurons and often co-expressed and widely distributed throughout the mammalian brain [50]. Their reduced expression suggests that inhibitory interneurons might be less frequent or less active in the brainstem of BSP-affected animals. One single cell colony that expresses *SST*, *NPY*, and *CCK* simultaneously is the basket cell, a common multipolar GABAergic inhibitory interneuron found in cortical structures of the cerebrum, cerebellum, and hippocampus. Basket cells target upper motor neurons [50]. In accordance with our findings, a previous histological study showed that the neurons in the red nucleus, reticular formation, and substantia nigra in the brains of BSP-affected cattle are smaller than in healthy animals [51]. To summarise, we concluded that it is very likely that molecular processes in the brainstem, as identified by DEGs in this study, are responsible for the pathogenesis of BSP.

The most striking DEG in all the examined neuromuscular axis tissues was *OOSP2*, which was manifold upregulated in the cases affected by BSP. OOSP2 is a secreted cytokine and activator of small GTPase-induced pathways that is mainly found in tissues of reproductive organs and belongs to the *PLAC1* gene family [52,53]. In mice, however, *OOSP2* was also found to be expressed to a lesser extent in other organs [52]. For example, the expression of *OOSP2* was detected in a population of antigen-presenting cells in the lung that elicit a strong type 2 T helper cell (T_H_2)-dominated immune response to viral infections [54]. These late-activator antigen-presenting cells (LAPCs) are therefore associated with exacerbated pathologies [54]. T_H_2-specific interleukins (IL4 and 10) have been found to cause severe post-infectious encephalitis in mice by attenuating the T_H_1-cellular immune response [55]. Interestingly, LAPCs specifically direct (Ag-activated) T-cells towards T-follicular-helper-cell (T_FH_-cells) differentiation [56]. T_FH_-cells are critical for B-cells to produce antibodies, and an unbalanced abundance of T_FH_-cells in relation to other regulators promotes autoreactivity [57]. Within a germinal center, the risk of autoantibody production is additionally associated with an excess of interleukin 21 (IL21), whose secretion from T_FH_-cells is regulated by a small GTPase-dependent pathway. Excessive IL21 has been found in at least seven autoimmune diseases to date [58]. It is noteworthy that IL21 also regulates various effectors within the cellular immune response and increases cytotoxicity [58]. Before to this study, neither the metabolic pathways nor the anatomical location of relevant pathologies in BSP were identified and we therefore applied whole tissue analyses. Whether the detected expression differences in brainstem tissue rely on immune cell immigration could best be validated in subsequent experiments by spatial transcriptomics [59].

In our study, we also found a downregulation of *SH2D3C* in the spinal cord. Interestingly this gene had previously been shown to be significantly downregulated in cervical spinal cord tissue [6] and was therefore a candidate gene. The encoded SH2 domain-containing protein 3C is an adapter protein that mediates cell signaling pathways and controls the immune response through chemotaxis. It is predominantly expressed by microglial cells and is an important activator of integrin-mediated T-cell adhesion and migration as well as tissue organization upstream of a small GTPase-induced intracellular pathway [60]. Its downregulation in the spinal cord could therefore be an indication of the migration of microglia towards the brain.

Since the RNA-Seq data presented here suggest that BSP may be due to an immunological process in the brainstem, we performed an immunohistochemical analysis using anti-Iba-1 and anti-CD3 antibodies. Iba-1 is a sensitive marker for microglia [61] and CD3 staining identifies T-cells [62]. Due to sampling at the slaughterhouse and the overall size of the bovine brainstem, section levels were not uniformly consistent between animals. Therefore, a clear topographical assignment of the histological and immunohistochemical findings was difficult, but they were found in both fiber tracts and nucleic areas. Microglial activation and T-cell infiltration were detected in both cohorts, but significantly more infiltrates were found in the brainstem sections of cattle affected by BSP. Limited by very few animals studied, these histological results can only sparsely encourage the theory that results from the transcriptomics part are due to immune cell immigration and significantly more animals must be examined to reliably confirm this theory.

The available data and results suggest that BSP is the result of a T_H_2-dominated immune response leading to an autoimmune reaction. This process is triggered by an *OOSP2*-overexpressing population of LAPCs leading to an accumulation of T_FH_-cells and excessive secretion of IL21 [54]. This leads to degeneration of the inhibitory interneurons of the brainstem and loss of control over the upper motor neurons, resulting in the classic BSP signs.

An interesting aspect of the results of the present study is that comparable processes are described in ALS. 90% of ALS cases and all cases of PLS cannot be linked to mutations in human genes. These cases are sporadic ALS [63]. Small GTPase-induced signaling pathways are detected in neurons and glial cells in ALS and other neurodegenerative diseases in humans and consequently lead to T-helper-cell-mediated hypersensitivity [64]. Several cytokines have been identified in ALS patients, suggesting that cytotoxic immune effector cells such as CD8 + T-cells (cytotoxic T-cells) and NK cells likely attack motor neurons and are responsible for the autoimmune-mediated pathogenesis of ALS in these patients [65]. Accordingly, we consider microglial cells to be the culprits in the expression of *OOSP2* and the triggers of the T-cell infiltrations detected in BSP. A juvenile-onset and slowly progressive form of ALS (ALS4) was found to be triggered by a cytotoxic T-cell response [66]. It is not yet clear whether cytotoxic T-cells are involved in the mechanism of BSP, but if this were the case, affected cattle could serve as models for ALS, as mild forms of ALS are comparable to BSP. Furthermore, although their biological functions differ between humans, mice, and cattle, members of the *PLAC1* gene family could be potential protein markers for immune-mediated ALS in humans.

The use of immunohistochemistry in the ALS rat model hSODG93A characterised inflammatory processes in the rodent brain as a CD4 + lymphocyte-dominated (T-helper cells) infiltration in the midbrain-interbrain-region, whereas CD8 + cell infiltrates (cytotoxic T-cells) were more restricted to the brainstem region and the blood-brain-barrier was impaired in areas of T-cell infiltrations [67]. Our immunohistochemical results suggest similar processes in the bovine brain.

## Conclusions

In summary, we found a link between pathological processes in the brainstem and BSP. This was indicated by a reduction in inhibitory neurotransmitters and an increased infiltration/activation of immune cells. There was no evidence that the spinal cord or peripheral nervous system is involved in the pathogenesis of BSP. However, upregulation of *OOSP2* was found in all neuromuscular tissues of animals affected by BSP. *OOSP2* is most likely expressed by LAPCs, suggesting that the animals have an active humoral adaptive immunity. This appears to be controlled by the excess of T_FH_-cells and leads to autoimmunity. Whether OOSP2 itself is involved in these processes remains unclear but it probably increases IL21 secretion via small GTPase-dependent pathways.

The presence of activated microglia and T-lymphocytes in the brainstem sections supports the idea of cerebral autoimmunity in BSP. The presumably affected cells are inhibitory interneurons, as the expression of important inhibitory neurotransmitters such as SST, NPY, and CCK was downregulated. This probably led to impaired modulation of upper motor neuron function, resulting in the specific symptoms of motor neuron disease in BSP.

We are aware that this study is merely hypothesising and that further mechanistic experiments are needed to definitively establish that BSP is an autoimmune-mediated disease. Subsequent studies need to confirm the link between OOSP2 expression and BSP in a larger number of animals. In addition, the causative immune cell population in cattle and the causative pathogens need to be defined. Spatial genomics would therefore be a promising tool and could eventually contribute to the development of a specific test for spastic paresis in cattle.

## Supporting information

S1 Fig Significantly enriched terms in gene ontology (GO) analysis. The Scatter plots illustrate the annotated GOs in biological process (BP), cellular component (CC), and molecular function (MF) in brainstem (A), spinal cord (B), and muscle (C) tissues. The vertical axis represents the enriched GOs and the horizontal axis represents the gene ratio (the ratio of differentially expressed genes enriched in each GO to the total number of genes in this GO term). The size and color of the dots indicate the number of genes and the range of *p*-value, respectively. The figure represents the top 5 significant GOs, if the number of enriched GOs in each category is more than this number.
(TIF)

S2 Video Illustration of two Holstein cows with signs of BSP in motion. (MOV)

S1 Table Candidate Gene list. (XLSX)

S2 Table DdPCR protocol and results. (XLSX)

S3 Table Complete list of enriched GOs. (XLSX)
