# Supplementary figures and images for: Findings from transcriptomics and immunohistochemistry indicate an autoimmune disease targeting brainstem inhibitory interneurons in bovine spastic paresis
Source: PLoS One. 2025 May 29;20(5):e0324633. doi: 10.1371/journal.pone.0324633 (PMC12121744; doi:10.1371/journal.pone.0324633)

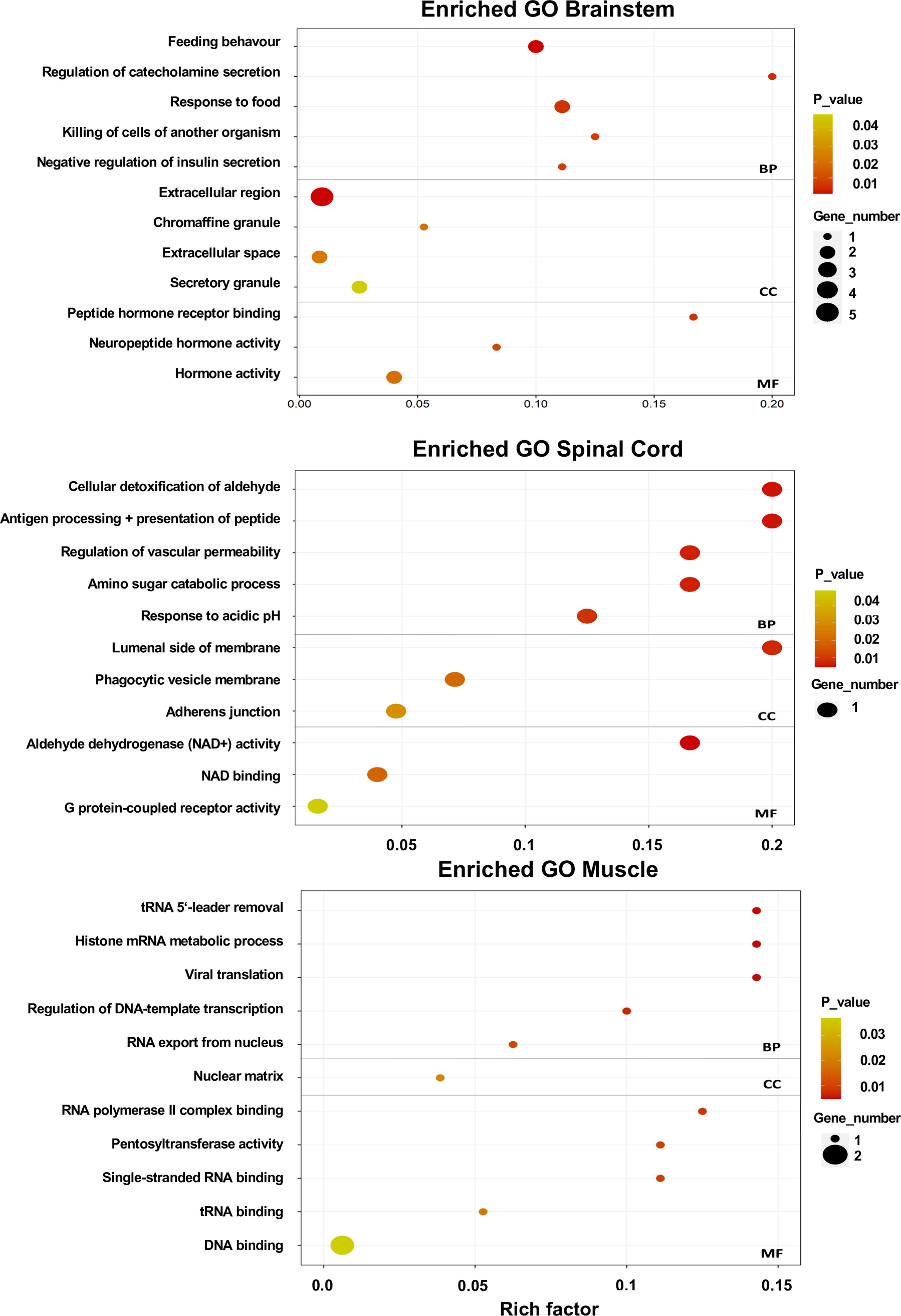

Supplement: S1 Fig — The Scatter plots illustrate the annotated GOs in biological process (BP), cellular component (CC), and molecular function (MF) in brainstem (A), spinal cord (B), and muscle (C) tissues. The vertical axis represents the enriched GOs and the horizontal axis represents the gene ratio (the ratio of differentially expressed genes enriched in each GO to the total number of genes in this GO term). The size and color of the dots indicate the number of genes and the range of p-value, respectively. The figure represents the top 5 significant GOs, if the number of enriched GOs in each category is more than this number. (TIF) [file pone.0324633.s001.tif]
